# *Pneumocystis jirovecii*-related spontaneous pneumothorax, pneumomediastinum and subcutaneous emphysema in a liver transplant recipient: a case report

**DOI:** 10.1186/s12879-019-3723-y

**Published:** 2019-01-18

**Authors:** Wong Hoi She, Kenneth S. H. Chok, Iris W. S. Li, Ka Wing Ma, Sui Ling Sin, Wing Chiu Dai, James Y. Y. Fung, Chung Mau Lo

**Affiliations:** 1 Department of Surgery, The University of Hong Kong, 102 Pok Fu Lam Road, Hong Kong, China; 2 State Key Laboratory for Liver Research, The University of Hong Kong, 102 Pok Fu Lam Road, Hong Kong, China; 3 School of Public Health, The University of Hong Kong, 102 Pok Fu Lam Road, Hong Kong, China; 4 Department of Medicine, The University of Hong Kong, 102 Pok Fu Lam Road, Hong Kong, China

**Keywords:** *Pneumocystis jirovecii*, *Pneumocystis* pneumonia, Post-liver transplant, Primary biliary cirrhosis

## Abstract

**Background:**

*Pneumocystis* pneumonia (PCP) is a common opportunistic infection caused by *Pneumocystis jirovecii*. Its incidence at 2 years or more after liver transplant (LT) is < 0.1%. PCP-related spontaneous pneumothorax and/or pneumomediastinum is rare in patients without the human immunodeficiency virus, with an incidence of 0.4–4%.

**Case presentation:**

A 65-year-old woman who had split-graft deceased-donor LT for primary biliary cirrhosis developed fever, dyspnea and dry coughing at 25 months after transplant. Her immunosuppressants included tacrolimus, mycophenolate mofetil, and prednisolone. PCP infection was confirmed by molecular detection of *Pneumocystis jirovecii*,in bronchoalveolar lavage. On day-10 trimethoprim-sulphamethoxazole, her chest X-ray showed subcutaneous emphysema bilaterally, right pneumothorax and pneumomediastinum. Computed tomography of the thorax confirmed the presence of right pneumothorax, pneumomediastinum and subcutaneous emphysema. She was managed with 7-day right-sided chest drain and a 21-day course of trimethoprim-sulphamethoxazole before discharge.

**Conclusion:**

Longer period of PCP prophylaxis should be considered in patients who have a higher risk compared to general LT patients. High index of clinical suspicion, prompt diagnosis and treatment with ongoing patient reassessment to detect and exclude rare, potentially fatal but treatable complications are essential, especially when clinical deterioration has developed.

## Background

*Pneumocystis* pneumonia (PCP) is a common opportunistic infection caused by *Pneumocystis jirovecii* affecting immunosuppressed patients with significant morbidity and mortality [1–3]. The incidence of PCP at 2 years or more after liver transplant (LT) is < 0.1% [2–4]. PCP-related spontaneous pneumothorax with or without pneumomediastinum is relatively uncommon in patients without the human immunodeficiency virus (HIV), with an incidence of 0.4–4% [5]. PCP co-infection with other viruses is occasionally seen and *cytomegalovirus* (CMV) is more common than with respiratory syncytial virus (RSV) and other viruses [2, 6, 7].

## Case presentation

**Fig. 1:**
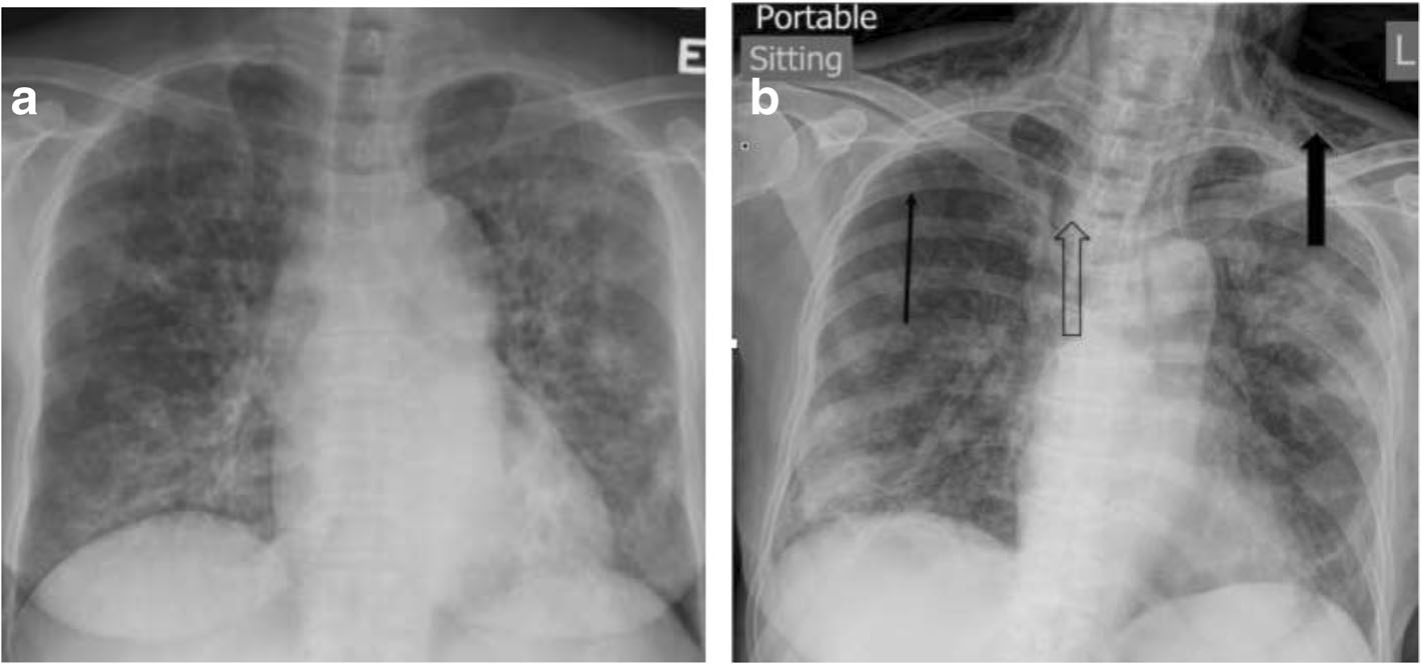
**a** Chest X-ray on admission showing bilateral peri-hilar interstitial infiltrates. **b** Chest X-ray showing right pneumothorax (thin arrow), subcutaneous emphysema (solid arrow) and pneumomediastinum (hollow arrow)

**Fig. 2:**
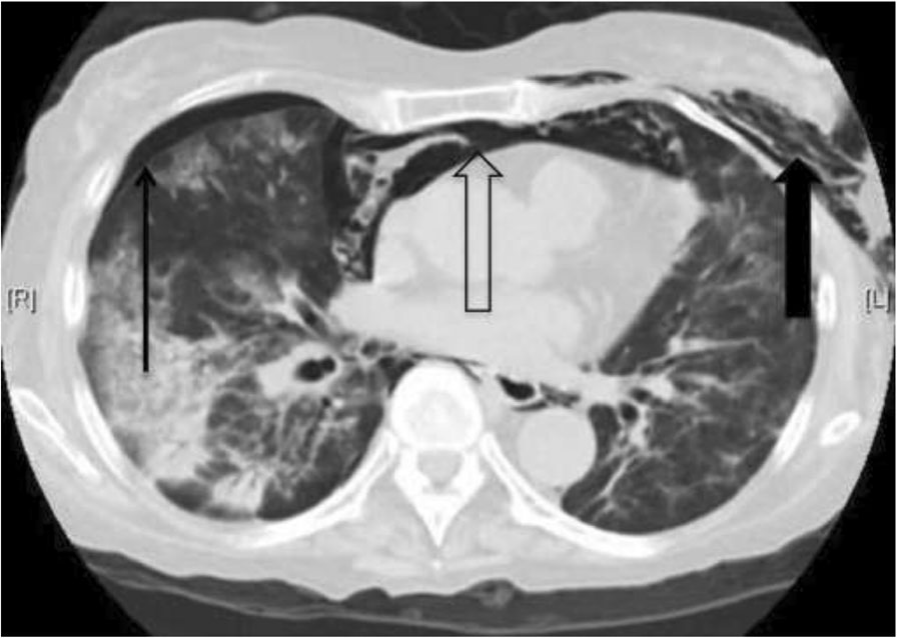
Computed tomography of thorax showing right pneumothorax (thin arrow), pneumomediastinum (hollow arrow), and subcutaneous emphysema (solid arrow)

**Fig. 3:**
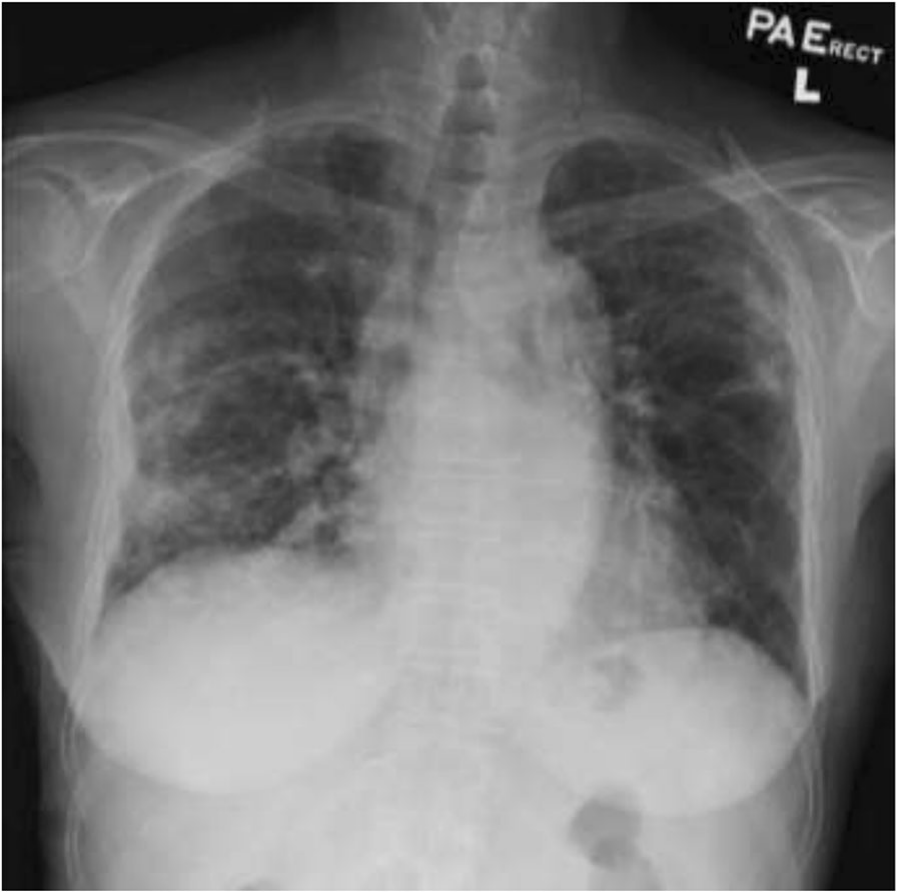
Chest X-ray on discharge showing resolution of the pneumothorax

A 65-year-old non-smoking and non-drinking woman had split-graft deceased-donor LT for end-stage primary biliary cirrhosis (PBC) (Tables 1 & 2). No pre-LT induction immunosuppressant was given. Her post-LT immunosuppressants included oral tacrolimus (1 mg twice daily) and mycophenolate mofetil (180 mg twice daily). She also had prednisolone (10 mg twice daily) immediately after LT and gradually tapered to 5 mg daily. Prophylactic medication included fluconazole (200 mg daily), trimethoprim-sulfamethoxazole (TMP-SMX) (480 mg daily) and acyclovir (400 mg tds) were also given for 3 months. She developed biliary anastomotic stricture and bile leakage, which improved with repeated endoscopic retrograde cholangiopancreatography with balloon dilatation without stenting. The last endoscopic retrograde cholangiopancreatography was performed at 22 months after LT. At 25 months after LT, she was admitted because of a 2-day history of fever, dyspnea and dry coughing. At admission, her blood pressure was 132/80 mmHg, pulse 106 beat per minute, and SpO2 88% at ambient air. SpO2 improved to 95% with supplemental oxygen (2 L/min) via nasal cannula, but rapidly deteriorated requiring 100% oxygen via re-breathing mask to maintain SpO2 ≥ 92%. Chest X-ray (Fig. 1a) and other investigations were performed (Tables 1 and 2). *Pneumocystis jirovecii*, CMV and RSV were detected in bronchoalveolar lavage by respective accredited in-house polymerase chain reaction. Her condition improved with intravenous TMP-SMX (trimethoprim component at 15 mg/kg/d divided in every 8 h), a tapering dose of corticosteroid for PCP and intravenous ganciclovir (5 mg/kg every 12 h as induction, followed by 5 mg/kg every 24 h as maintenance) for CMV. Her immunosuppressants were reduced and tapered during the PCP treatment. On day-10 TMP-SMX, her chest X-ray showed subcutaneous emphysema bilaterally and right pneumothorax suspected of pneumomediastinum (Fig. 1b). Computed tomography of the thorax confirmed the presence of right pneumothorax, pneumomediastinum and subcutaneous emphysema (Fig. 2). She was managed with 7-day chest drain in situ with a standard Argyle-type chest tube of Fr-32 until her right lung re-expanded, in addition to 21-day TMP-SMX. She was not put on mechanical ventilation. She survived and was discharged on day 31 after admission. Chest X-ray on discharge showed resolution of the pneumothorax (Fig. 3).

**Table 1:** Results of investigation performed for primary biliary cirrhosis and liver transplant workup

Test performed	Results (Reference range)
Pre-transplant	
Hepatitis serology	
HBsAg	Negative
Anti-HBs	< 10 mlU/mL
Anti-HBc (total)	Negative
Anti-HAV (total)	Positive
Anti-HAV IgM	Negative
Autoimmune markers	
C3	135 (76–150) mg/dL
C4	26 (9–35) mg/dL
ANA titre	Could not be interpreted due to cytoplasmic staining
Anti-ds DNA	7 (0–35) IU/ml
Anti-smooth muscle Ab	Negative
Anti-mitochrondrial Ab	Positive, M2 pattern
Immunoglobulin (Ig) pattern	
IgG	3540 (819–1725) mg/dL
IgA	407 (70–386) mg/dL
IgM	474 (55–307) mg/dL
Lung function test	
Forced vital capacity (FVC)	2.23 L, 96%
Forced expiratory volume during the 1 sec/FVC (FEV1/FVC)	70% predicted
liver biopsy – hepatectomy	Primary biliary cirrhosis with impending cirrhosis
This admission	
Blood culture	No growth
Nasopharyngeal aspirate	Polymerase chain reaction (PCR) positive for *Respiratory syncytial virus* (RSV) and *Enterovirus(EV)/ Rhinovirus (RV)*
	Negative for *Influenza A virus*, *Influenza B virus*, *Parainfluenza virus*, *Adenovirus*, *human metapneumovirus*
Sputum culture	*Candida albicans*
Mid-stream/Catheterized urine culture	No growth
Serum Cryptococcal antigen	Negative
Plasma CMV pp65 Antigen	Negative
Bronchoalveolar lavage fluid	
- Gram stain	No organism seen
- Bacterial culture	Candida species
- Acid fast bacilli (AFB) smear and culture	Negative and no growth of AFB
- Fungal smear / culture	Smear negative / Candida albicans
- TB-PCR	Negative
- *Pneumocystis jirovecii* qualitative PCR	Positive
- CMV qualitative PCR	Positive
- Respiratory viruses antigen detection by immunofluorescence	Positive for RSV
- Respiratory viruses qualitative PCR	Positive for RSV
	Negative for EV/RV, *Influenza A virus*, *Influenza B virus, Parainfluenza virus, adenovirus, human metapneumovirus*
Liver biopsy (28 months post-transplant)	Non-specific change

*Ab* antibody, *ANA* anti-nuclear antibody, *Anti-Hbc* antibody to hepatitis B core antigen, *CMV* cytomegalovirus, *ds* double-stranded, *HAV* hepatitis A virus, *HBsAg* hepatitis B surface antigen, *IgM* immunoglobulin M, *NA* not available, *TB* tuberculosis

**Table 2:** Profiles of laboratory investigations

	Pre-transplant	Day of transplantation	24 months post-transplant	Day of hospital admission	Day of pneumothorax confirmed	Day of discharge from hospital	30 months post-transplant	Reference range
Complete blood picture								
white blood cell (wcc, ×10^9^/L)	3.15	6.82	8.97	11.92	22.61	6.91	4.53	3.89–9.93
Neutrophil (×10^9^/L)	2.30	2.93	8.40	10.51	19.76	4.69	3.17	2.01–7.42
Lymphocyte (× 10^9^/L)	0.50	0.75	0.43	0.63	1.93	1.66	1.05	1.06–3.61
Monocyte (×10^9^/L)	0.16	0.22	0.14	0.69	0.83	0.21	0.26	0.18–0.65
Hemoglobin (Hb, g/dL)	10.1	9.8	10.6	11.6	10.1	9.3	11.3	11.5–14.8
Platelet (Plt, ×10^9^/L)	116	121	114	265	352	209	126	154–371
Clotting profile								
PT (sec)	13.6	34.8	13.2	13.1	13.3	12.5	12.9	
INR	1.2	3.1	1.1	1.1	1.1	1.0	1.1	
APTT (sec)	30.3	> 110.0	25.7	29.1	26.1	22.5	26.8	
Renal function test								
Sodium (Na, mmol/L)	141	153	139	140	129	139	143	136–148
Potassium (K, mmol/L)	3.7	4.1	4.7	4.6	4.0	3.5	5.0	3.6–5.0
Urea (mmol/L)	6.1	9.4	13.4	12.7	14.6	1.7	15.0	2.9–8.0
Creatinine (umol/L)	62	63	94	158	96	56	80	49–82
Liver function test								
Total protein (g/L)	80	27	66	71	57	62	86	67–87
Albumin (Alb, g/L)	29	< 10	40	43	30	32	44	39–50
Globulin (Glo, g/L)	51	19	26	28	27	30	42	26–40
Bilirubin, total (μmol/L)	126	127	7	21	5	7	8	4–23
Alkaline phosphatase (ALP, U/L)	264	41	91	92	116	145	115	47–124
Alanine transaminase (ALT, U/L)	106	1025	57	30	38	52	63	7–36
Aspartate transaminase (AST, U/L)	147	2339	26	24	63	47	50	14–30
Gamma-glutamyl transferase (GGT, U/L)	100	42	68	45	NA	NA	101	Up to 35
Arterial blood gas	NA	NA	NA		NA	NA	NA	
FiO2				21%				
pH				7.43				7.35–7.45
pCO_2_ (kPa_)_				4.0				4.7–6.0
pO_2_ (kPa)				12.7				10.6–14.0
Bicarbonate (mmol/L)				19				22–26
Base excess (mmol/L)				−4				−4 - + 2

*NA* not available

## Discussion

This case demonstrated PCP-related pneumothorax, pneumomediastinum and subcutaneous emphysema at 22 months after PCP prophylaxis in a recipient of LT for end-stage PBC at 25 months after transplant.

*Pneumocystis jirovecii* (previously *Pneumocystis carinii*) is considered a distinct species causing human PCP infection [8]. It is a yeast-like fungus of the genus *Pneumocystis*. It is an opportunistic agent affecting immune-compromised hosts. PCP is one of the conditions that define Acquired Immune Deficiency Syndrome (AIDS). It is more commonly seen in other immunosuppressed patients due to underlying diseases, immunosuppressants or chemotherapy [3].

The reported incidence of PCP in LT recipients without PCP prophylaxis is 3–11%, which is the lowest among solid organ and bone marrow transplant recipients [2–4]. Most of the cases developed during the initial 6 to 9 months and > 70% developed within 12 months of LT, but the development could be as early as within 1 to 3 weeks of LT [1, 4]. PCP prophylaxis is recommended for 6–12 months post-transplant [9]. In LT recipients with immediate post-LT 6-month TMP-SMX as PCP prophylaxis, the incidence of PCP was reported to be 0.3% at 6 months, 0.6% at 6–12 months, 0.9% at 1 year, and < 0.1% at more than 1 year [1], and the incidence at 12 months after LT was reported to be 0.24 per 1000 persons per transplant year [4].

The risk factors for PCP in our patient was prolonged use of prednisolone and simultaneous use of tacrolimus and mycophenolate mofetil, resulting in a reduction of CD4 T-cells (CD4) [10]. Although mycophenolate mofetil may have intrinsic activity against *Pneumocystis jirovecii*, tacrolimus has been found to boost the growth of *Pneumocystis jirovecii* in vitro [11, 12]. In addition to lympholytic activity of steroid, the resultant depressed cell-mediated immunity rendered our patient susceptible to opportunistic infections [13]. In earlier reports, corticosteroid use and depressed cell-mediated immunity were significant risk factors for PCP in non-HIV patients, irrespective of the use of mechanical ventilation [14]. In non-HIV patients with PCP, > 80% had prednisolone use at ≥20 mg/day for ≥1 month, and 25% had corticosteroid as a monotherapy immunosuppressant. LT recipients without PCP prophylaxis developed PCP even just on prednisolone at 5 mg/day to 20 mg/day [3, 4, 14]. Our patient received oral prednisolone at 5 mg/day for 25 months, lower than the median dose of 41.8 (22.3–61.5) mg/day but longer than the duration of corticosteroid use of 4.8 (1.8–10.1) months reported in a cohort of non-HIV patients with PCP who required mechanical ventilation, including 12.5% of LT recipients [6].

Moreover, the depressed cell-mediated immunity also resulted in latent CMV reactivation, which also acted as immunosuppressive agent suppressing T-helper and antigen-presenting cells’ functions [13]. The clinical significance of compartmentalized CMV reactivation in bronchoalveolar lavage might be controversial but should not be overlooked, given our patient’s clinical context. In non-HIV patients with PCP who required mechanical ventilation, 29 and 2.1% of the respiratory specimens were found to be PCP with CMV or RSV co-infection respectively [6]. A significantly higher rate of PCP with co-infection has been found in non-surviving non-HIV patients than those surviving [10]. Thirdly, our patient’s PBC might be related to depressed cell-mediated immunity. PBC patients have a significantly lower level of circulating CD4 than healthy persons, irrespective of simultaneous alveolitis [15], and have detectable mitochondrial antigen-specific-CD4 in peripheral blood, liver-drainage lymph nodes and liver, which is not found in healthy or other liver-diseased persons [16]. Hence, PBC may be a systemic autoimmune disease affecting lungs and/or other organs, apart from its well-established tissue-specificity targeting intrahepatic biliary epithelial cells [17]. We did not check the CD4 level of our patient, as this is currently used only in the HIV patients. Our patient’s pre-LT total lymphocyte count of 500 cells/μL (Table 2) predicted a CD4 level of < 200 cells/μL [18]. Furthermore, she received steroid for PBC-associated autoimmune disease and anti-rejection immunosuppressants, which rendered her susceptible to PCP. Unfortunately, she could not receive long-term PCP prophylaxis due to underlying renal impairment.

Her acute 2-day symptoms were concordant with the shorter symptom duration (20% in < 3 days, or ~ 5 to 6 days) in non-HIV patients with PCP, as compared to sub-acute and longer symptom durations (25 to 28 days) observed in HIV patients with PCP [3, 13, 14]. She survived despite the high mortality of PCP: > 80% in adult LT recipients, and 30–40% in non-HIV patients, especially those who have solid organ tumors or acute respiratory distress syndrome or require invasive mechanical ventilation (comparing to 10–20% in HIV PCP) [2, 3, 13].

Our patient presented with pneumonia (which is the commonest primary manifestation of *Pneumocystis jirovecii* infection in non-HIV patients with PCP [2, 3, 14] and typical diffuse interstitial and intra-alveolar infiltrates on chest X-ray [3]. She had a rare condition: PCP-related spontaneous pneumothorax, pneumomediastinum and subcutaneous emphysema without prior or subsequent use of mechanical ventilation. The higher incidence of spontaneous pneumothorax in AIDS patients with PCP than those without (up to 9% vs 0%) is related to PCP [17]. In previous studies, up to 9% of hospitalized HIV patients with PCP developed pneumothorax [19, 20], but only 2–4% of non-HIV patients with PCP did [3]. In another cohort of non-HIV patients with PCP who required mechanical ventilation (including 35% solid organ transplant recipients), 19% developed pneumothorax [6]. In another study of HIV patients, 20% of those with PCP developed pneumothorax [19].

The pneumothorax in our patient was likely due to rupture of necrotic lung tissues due to proteases released from activated macrophages and/or alveolar over-distention secondary to bronchiolar inflammation caused by *Pneumocystis jirovecii* [5, 17]. In previous studies, preceding sub-pleural necrosis, pulmonary cysts or pneumatoceles, and bleb formation with wall thickness thinning occurred in 3–6% of non-HIV patients with PCP [3, 17]. Neutrophilic lung inflammation may cause pneumothorax [10]. Lower *Pneumocystis jirovecii* inoculum found in non-HIV patients with PCP may suggest direct invasion or toxicity by the micro-organism as pathogenic mechanism is less substantiated [3, 13]. Significantly higher neutrophil and lower lymphocyte percentages have been found in bronchoalveolar lavage of non-surviving non-HIV patients with PCP [10]. In a study by Krowka et al., 40–90% of PBC patients had subclinical fibrosing alveolitis with reduced diffusion capacity of carbon monoxide, which was associated and correlated with PBC clinical and histological severity [21]. Gas exchange abnormality attributed to hypoxaemia and increased breathing efforts may be conducive to pneumothorax. In PBC, it is uncertain but possible that the characteristic CD4 and activated alveolar macrophages predominating inflammatory infiltration would exaggerate pneumatocele wall thinning, which is conducive to pneumothorax [22].

Pneumomediastinum likely results from air leakage from pneumatoceles and tracks along the bronchovascular tissue sheath into the hilum and mediastinum. Mucosal break of the esophagus or tracheobronchial tree with presence of gas-producing micro-organisms may cause pneumomediastinum, with progress involving pericardium [23]. Our patient’s conditions were unlikely iatrogenic. Her previous endoscopic retrograde cholangiopancreatography was performed 3 months before her admission. No transbronchial biopsy was taken during fiberoptic bronchoscopy, and no inhaled pentamidine or mechanical ventilation was used [5].

Since CD4 is the main target of HIV, it can be used as a specific marker for PCP prophylaxis threshold in HIV management. In previous studies, the median CD4 level in non-HIV patients at PCP diagnosis was 61 to 329 cells/μL, which was higher than the level in HIV patients (< 50 cells/μL), and > 35% of these non-HIV patients had CD4 > 300 cells/μL [3, 14, 24, 25]. On the other hand, HIV patients with current or previous PCP and pneumothorax had CD4 <  100 cells/μL, and 58% of them had CD4 < 50 cells/μL [25]. At our center, CD4 check was not routinely performed for non-HIV patients. Our patient’s total lymphocyte count of 630 cells/μL at admission (Table 2) predicted a CD4 level of < 200 cells/μL [18]. Clinical parameters as independent predictors of PCP in non-HIV patients include serum albumin < 28 g/L, PaO2/FiO2 ratio < 210, and CD3 < 625 cells/μL [14]. Our patient had no chronic lung disease, simultaneous bacteremia, or high blood urea nitrogen, which are independent predictors for mortality of non-HIV patients with PCP [10]. Other mortality predictors include CD8 < 160 × 10^6^/L and PaO2/FiO2 ratio < 160 [14].

The first-line PCP prophylaxis is TMP-SMX. Alternatives include atovaquone, pyrimethamine, sulfadoxine and dapsone [3]. There are no guidelines on PCP prophylaxis duration for LT recipients. With different centers having different practice, balancing the incidence of PCP and the side effects of medication, the duration varies between 1 and 12 months, or is an indefinite period [4]. Different criteria for routine or targeted post-LT PCP prophylaxis are based on adult incidence rate ≥ 3.5%, anytime total lymphocyte count < 400 cells/μL, anti-rejection immunosuppressant use, till prednisolone dose < 10 mg/day, or the presence of 2 or more of the following risk factors: pretransplant renal failure, fulminant hepatic failure, retransplantation, intraoperative Roux-loop-hepaticojejunostomy, post-LT intensive care unit stay > 5 days, renal failure, albumin-creatinine ratio, and total parenteral nutrition > 48 h. Sarwar et al. [4] found that calcineurin inhibitor-based immunosuppressant monotherapy could reduce the risk of PCP after LT without prophylaxis. At our center, immediate post-LT 3-month oral TMP-SMX or monthly inhalation pentamidine is given, depending on the patient’s renal function and glucose-6-phosphate dehydrogenase status. The interaction between tacrolimus and mycophenolate mofetil could result in an excessive degree of immunosuppression [26, 27]. The occurrence of PCP was found to be associated with an increased amount of immunosuppression, such as combination of tacrolimus, mycophenolate mofetil, and corticosteroid [28]. Hence, in patient who may require multiple immunosuppressive agents, longer period of PCP prophylaxis should be considered.

The standard PCP treatment is 3-week oral or intravenous TMP-SMX. For TMP-SMX intolerance or non-responsiveness, intravenous pentamidine and combined oral primaquine and intravenous clindamycin are two alternatives [3]. Use of adjuvant corticosteroids in non-HIV patients with PCP with moderate-to-severe severity (PaO_2_ < 70 mmHg at room air) is controversial, although such use has been well established in AIDS patients [3, 10]. Ongoing assessment for causes of clinical deterioration is crucial for prompt management (and intensive care unit monitoring, if necessary), which is critical in reducing mortality [6].

## Conclusion

PCP in LT recipients has abrupt symptom onset with rapid progression and carries a high mortality rate. Longer period of PCP prophylaxis should be considered in patients who have a higher risk compared to general LT patients. PCP-related pneumothorax rarely occurs in LT recipients. However, vigilance in detecting and excluding rare, potentially fatal but treatable complications is crucial, especially when clinical deterioration has occurred.

## Abbreviations

AIDS: Acquired immune deficiency syndrome
CD4: CD4 T-cells
CMV: *Cytomegalovirus*
HIV: Human immunodeficiency virus
LT: Liver transplant
PBC: Primary biliary cirrhosis
PCP: *Pneumocystis* pneumonia
RSV: Respiratory syncytial virus
SMX: Sulphamethoxazole
TMP: Trimethoprim
